# Supplementary figures and images for: Proteins that carry dual targeting signals can act as tethers between peroxisomes and partner organelles
Source: PLoS Biol. 2024 Feb 20;22(2):e3002508. doi: 10.1371/journal.pbio.3002508 (PMC10906886; doi:10.1371/journal.pbio.3002508)

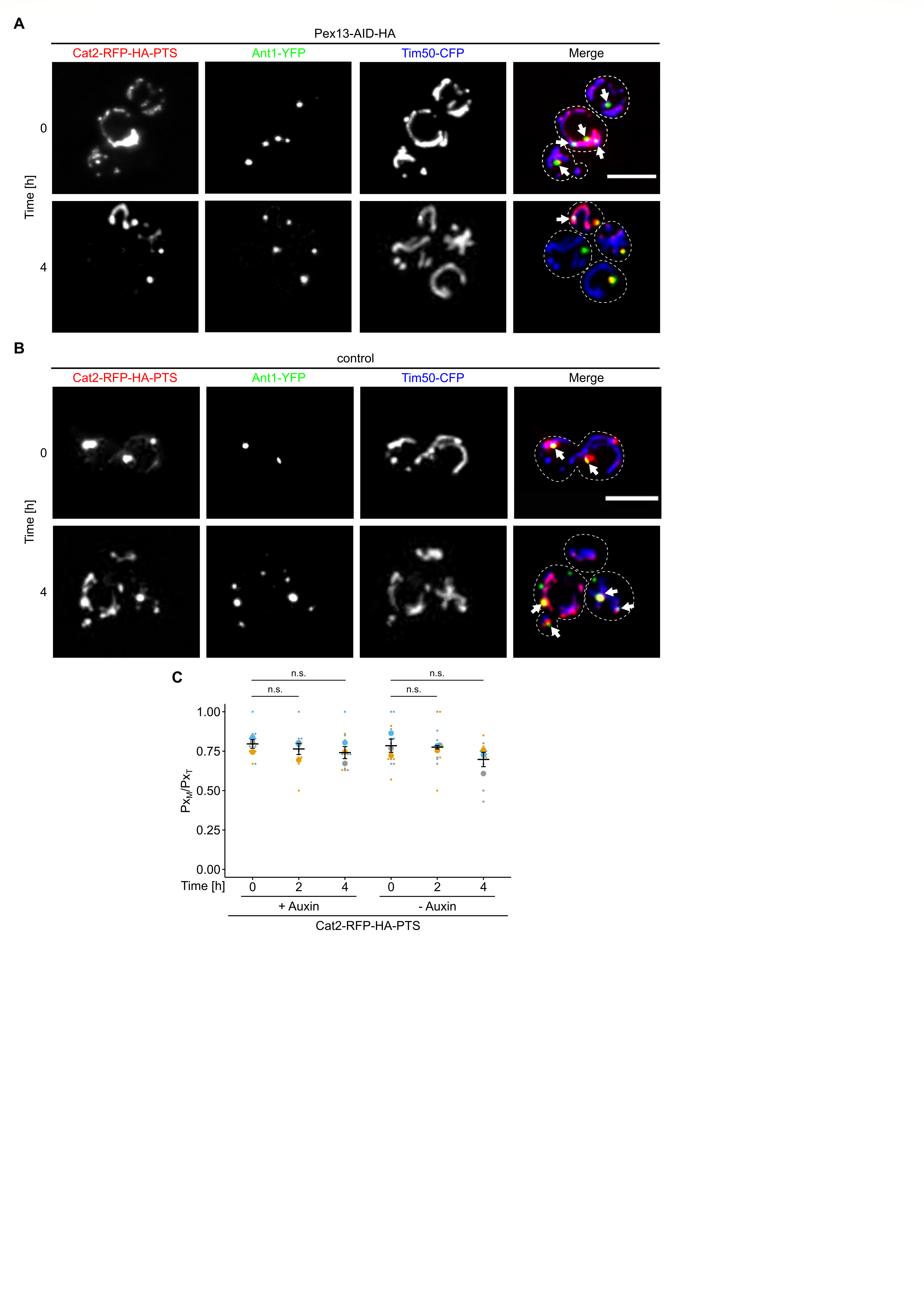

Supplement: S5 Fig — (A and B) Images are from indicated strains expressing Ant1-YFP (green), Tim50-CFP (blue), and Cat2-RFP-HA-PTS (red) photographed at indicated time points after addition of 2 mM indole-3-acetic acid (auxin). Arrows indicate peroxisomes that contact mitochondria. (C) Quantifications of contact in control cells lacking Pex13-AID (shown in B) are based on n = 3 experiments. Each color represents 1 experiment. Error bars represent standard error of the mean. P-values were calculated with a one-way ANOVA combined with a Tukey test. Underlying data for quantifications can be found in S1 Data. (TIFF) [file pbio.3002508.s005.tiff]

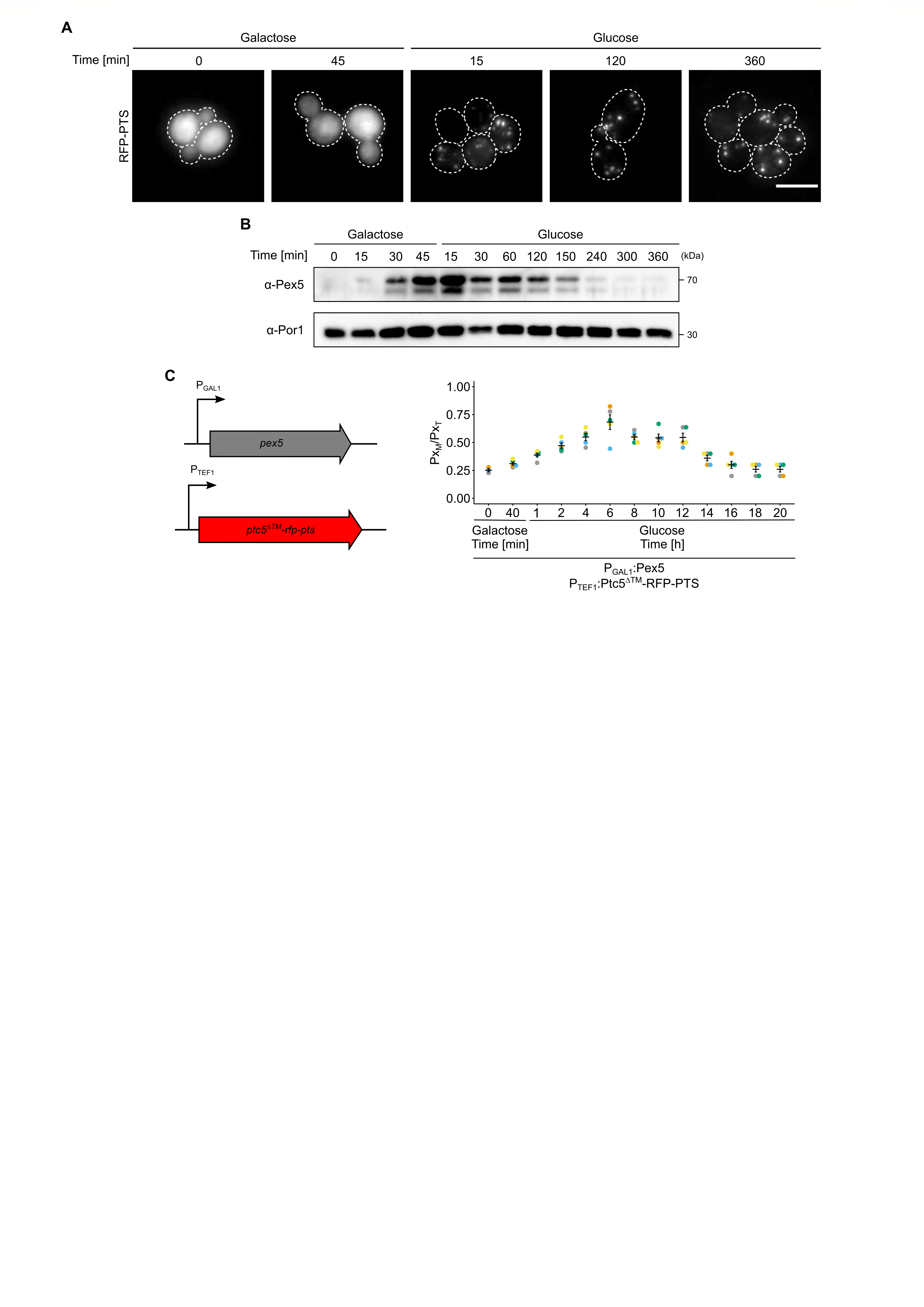

Supplement: S6 Fig — (A) Fluorescence microscopic images of a galactose chase and shut-off experiment using a galactose-inducible conditional pex5 mutant expressing RFP-PTS. Scale bars represents 5 μm. (B) Pex5 levels from indicated time points of a strain expressing Tim50-CFP, Ant1-YFP, and Ptc5ΔTM-RFP-PTS were analyzed by SDS-PAGE and immunoblot. Por1 served as loading control. (C) Quantifications of PerMit contacts in the course of the galactose chase and shut-off experiment. Note that this experiment was carried out via automated time-lapse imaging and does not directly reflect the Pex5 concentrations shown in S5B Fig, which were obtained from incubations in liquid media. Underlying data for quantifications can be found in S1 Data. (TIFF) [file pbio.3002508.s006.tiff]

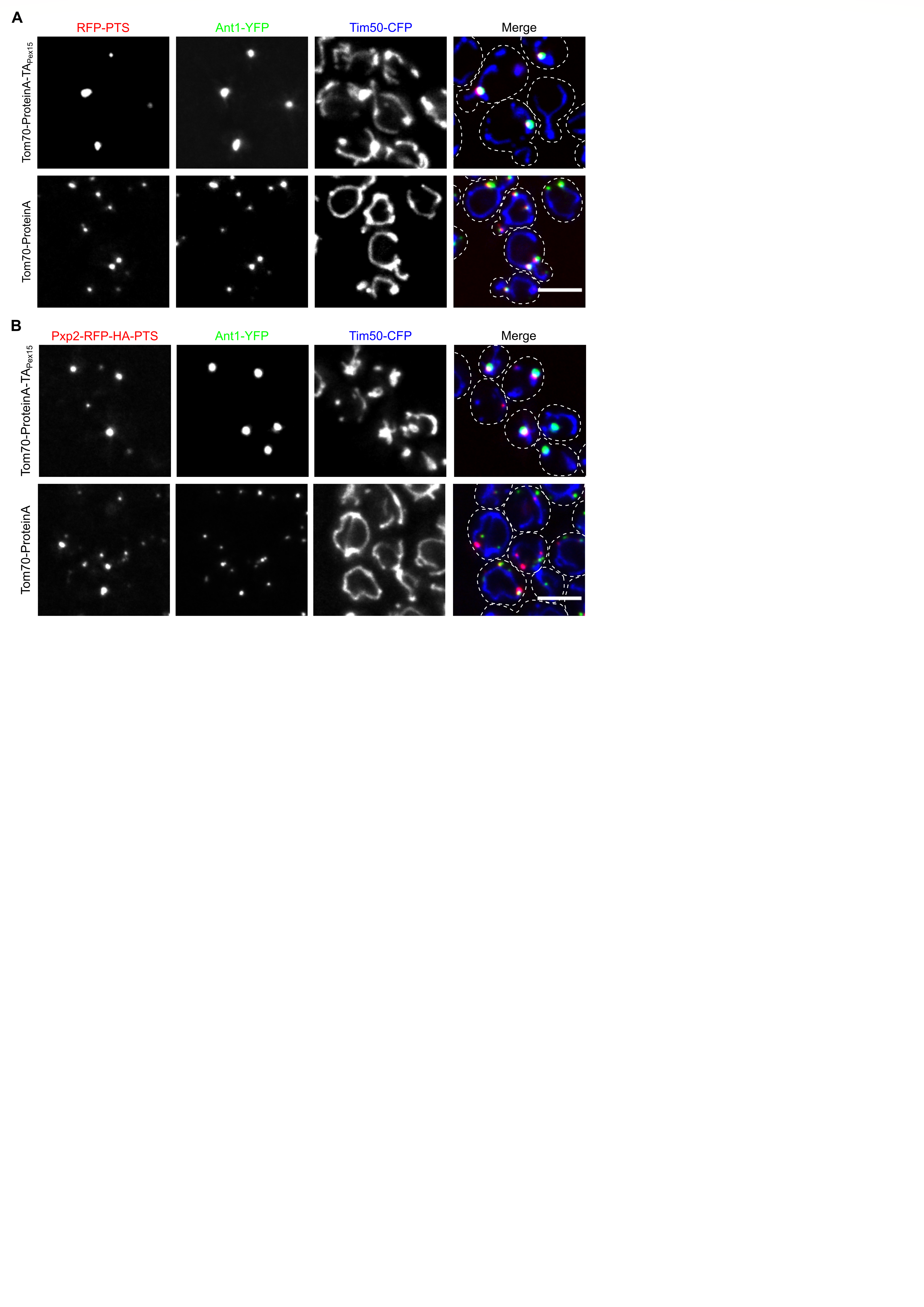

Supplement: S14 Fig — Fluorescence microscopic images of cells co-expressing the peroxisomal marker RFP-PTS (red, A) or Pxp2-RFP-PTS (red, B), the peroxisomal membrane protein Ant1-YFP (green), and the mitochondrial protein Tim50-CFP. Scale bars represent 5 μm. (TIFF) [file pbio.3002508.s014.tiff]

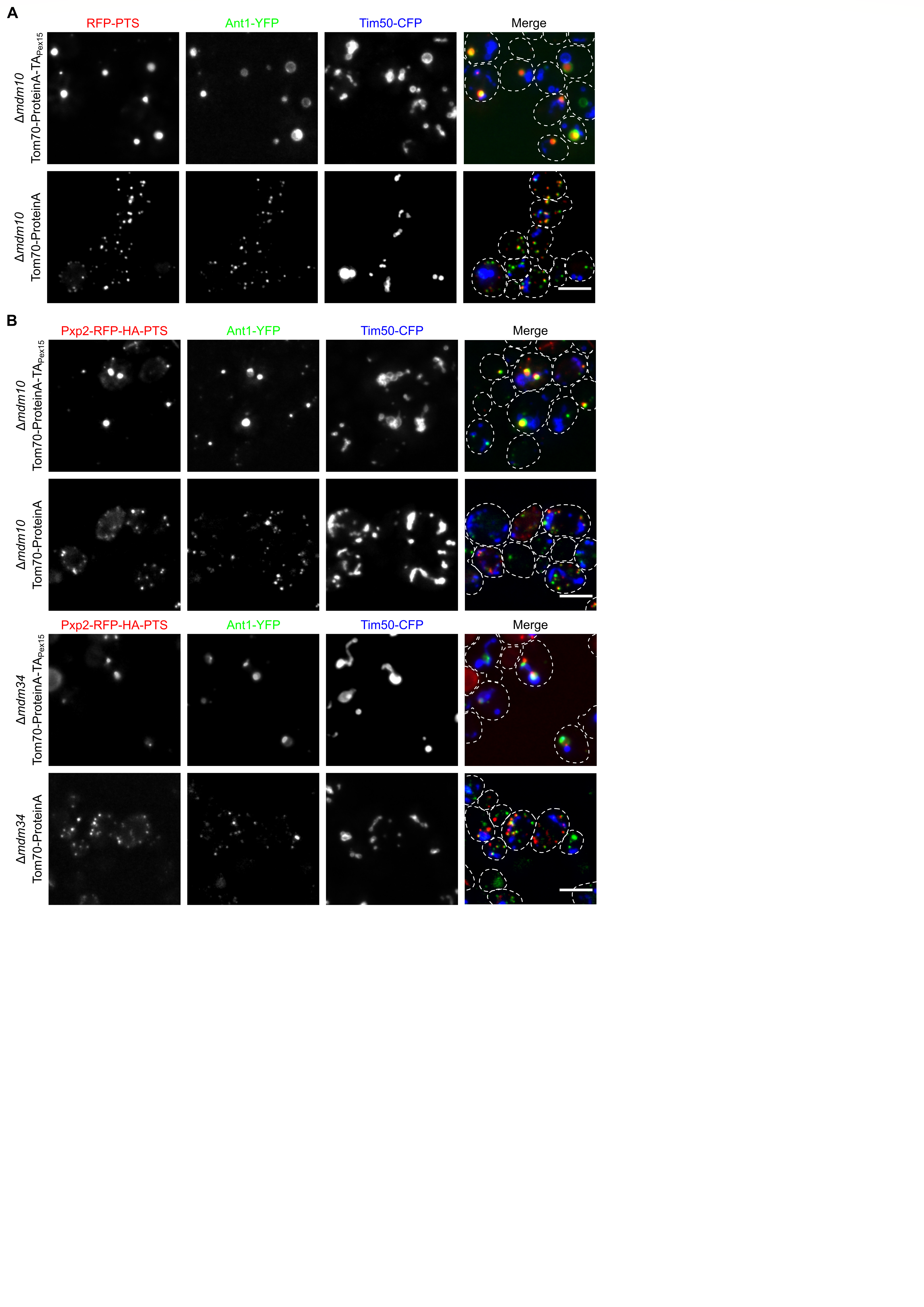

Supplement: S15 Fig — Images of strains deleted for mdm10 or mdm34 co-expressing RFP-PTS (A, red) or Pxp2-RFP-PTS (B, red), the peroxisomal membrane protein Ant1-YFP (green), and the mitochondrial protein Tim50 (blue) either in the presence of Tom70-ProteinA-TAPex15 or a control protein. Scale bars represent 5 μm. (TIFF) [file pbio.3002508.s015.tiff]

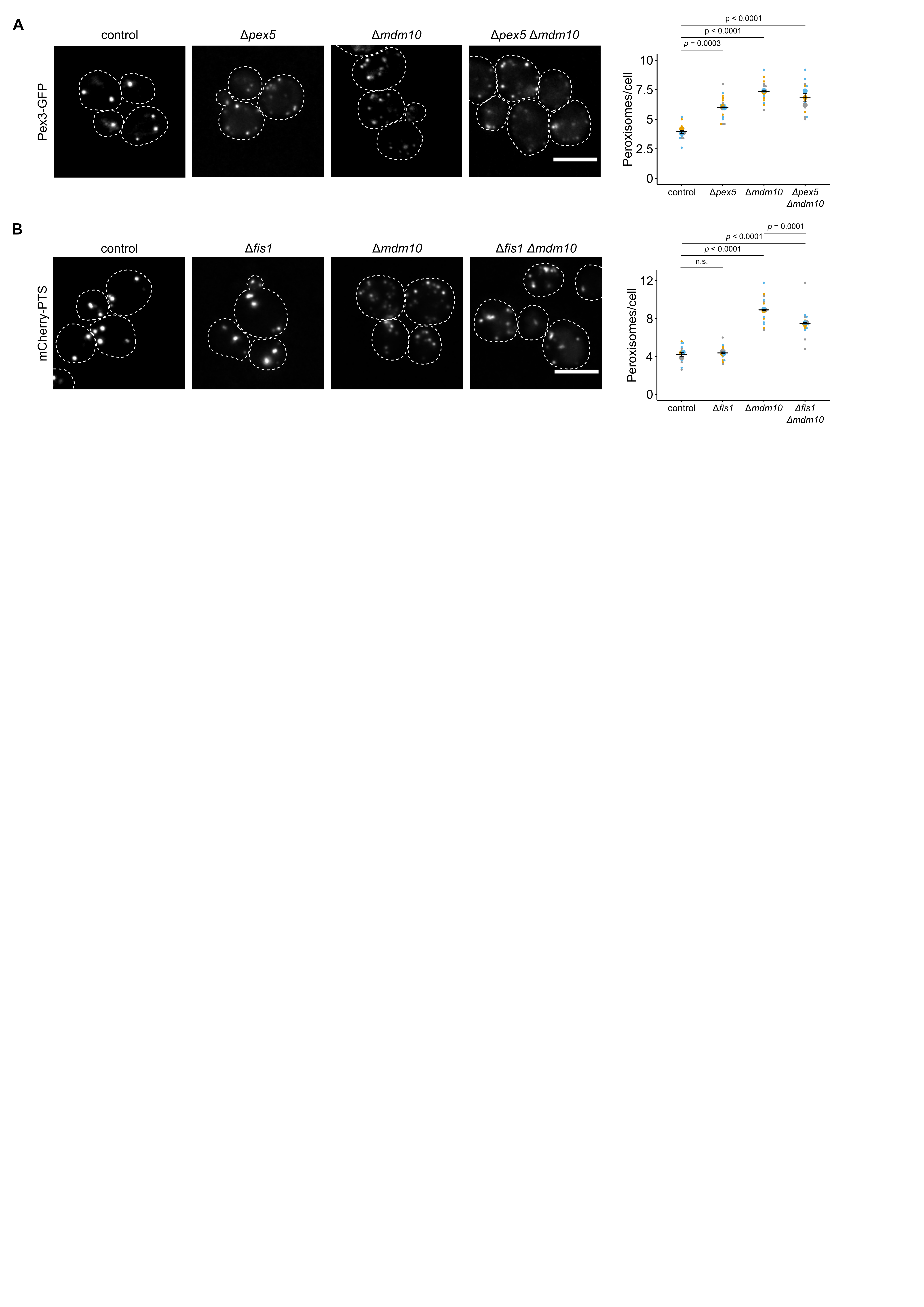

Supplement: S16 Fig — (A) Indicated mutants expressing Pex3-GFP were analyzed by fluorescence microscopy (left). Quantifications of peroxisome number (right). (B) Indicated mutants expressing mCherry-PTS were analyzed by fluorescence microscopy (left). Quantifications of peroxisome number (right). Scale bars represent 5 μm. Quantifications are based on n = 3 experiments. Each color represents 1 experiment. Error bars represent standard error of the mean. P-values were calculated with a one-way ANOVA combined with a Tukey test. Underlying data for quantifications can be found in S1 Data. (TIF) [file pbio.3002508.s016.tif]

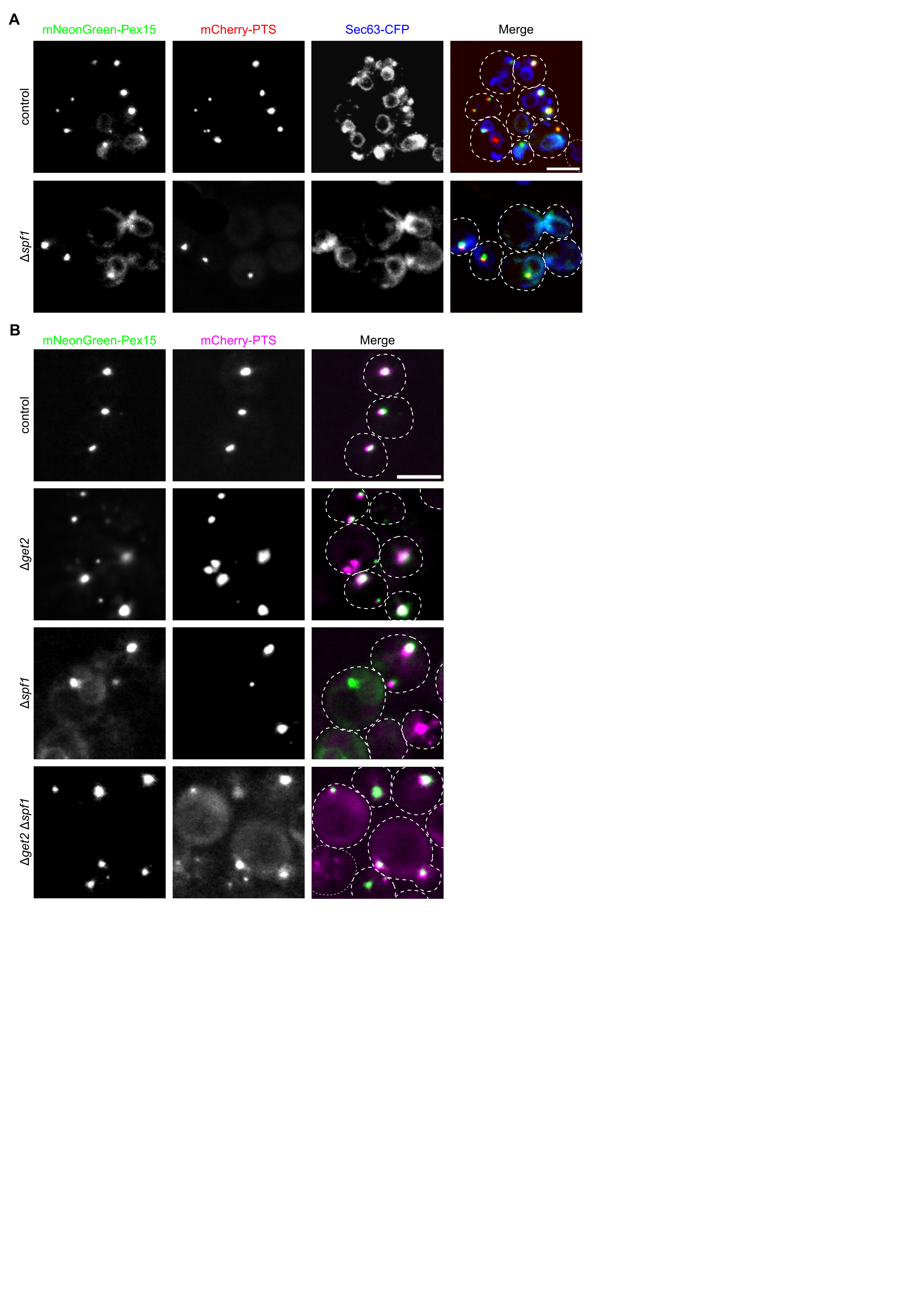

Supplement: S17 Fig — (A) Fluorescence microscopic images of indicated strains expressing mNeonGreen-Pex15 (green), mCherry-PTS (magenta), and Sec63-CFP (blue). (B) Fluorescence microscopic images indicated strains expressing mNeonGreen-Pex15 (green), mCherry-PTS (magenta), and Tim50-CFP (blue). (C) Fluorescence microscopic images of control and indicated mutant cells expressing mNeonGreen-Pex15 (green) and mCherry-PTS (magenta). Scale bar represents 5 μm. (TIF) [file pbio.3002508.s017.tif]
