# Supplementary material for: Proteins that carry dual targeting signals can act as tethers between peroxisomes and partner organelles
Source: PLoS Biol. 2024 Feb 20;22(2):e3002508. doi: 10.1371/journal.pbio.3002508 (PMC10906886; doi:10.1371/journal.pbio.3002508)
Supplement: S1 Raw Images — (PDF) [file pbio.3002508.s028.pdf]

Blot 1: Figure 2F (left)

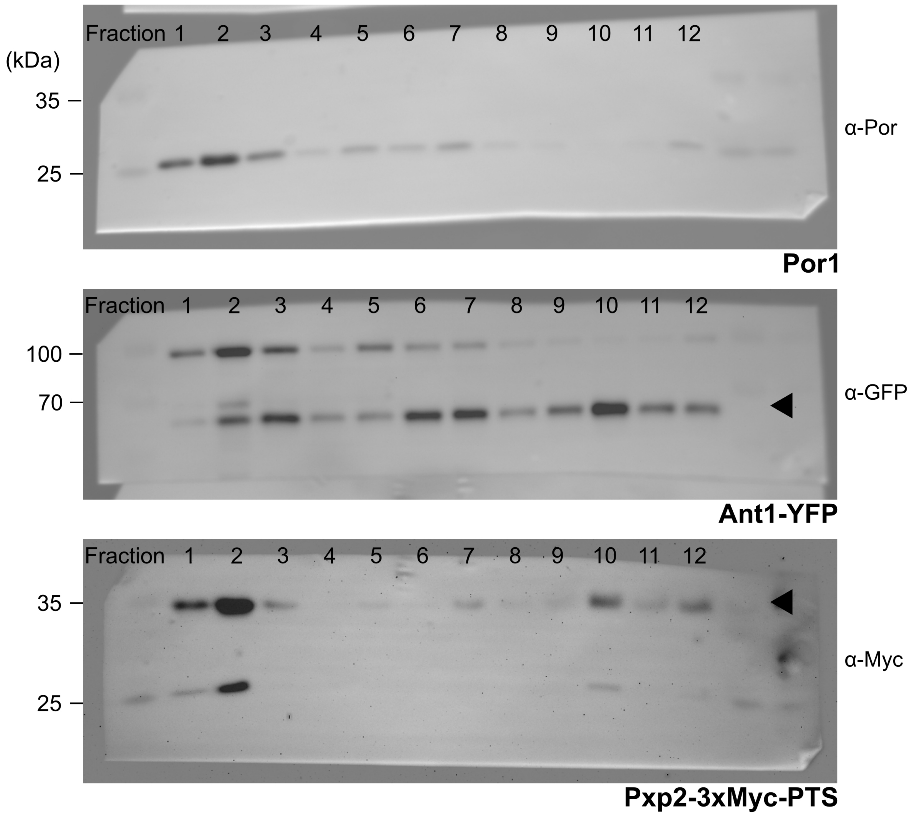

Blot 2: Figure 2F (right):

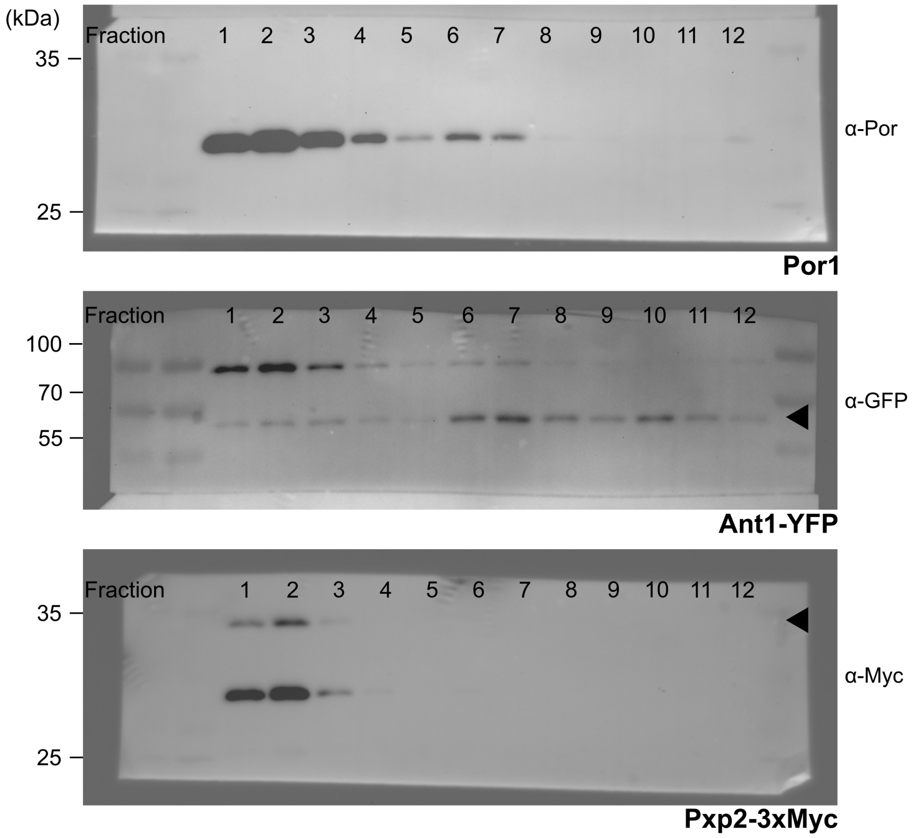

Blot 3: Figure 3F

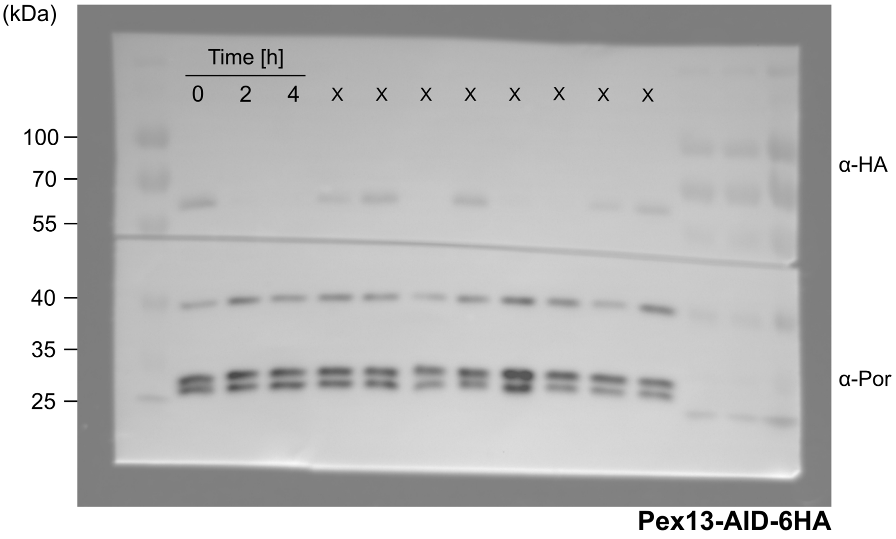

Blot 4: Figure 4E

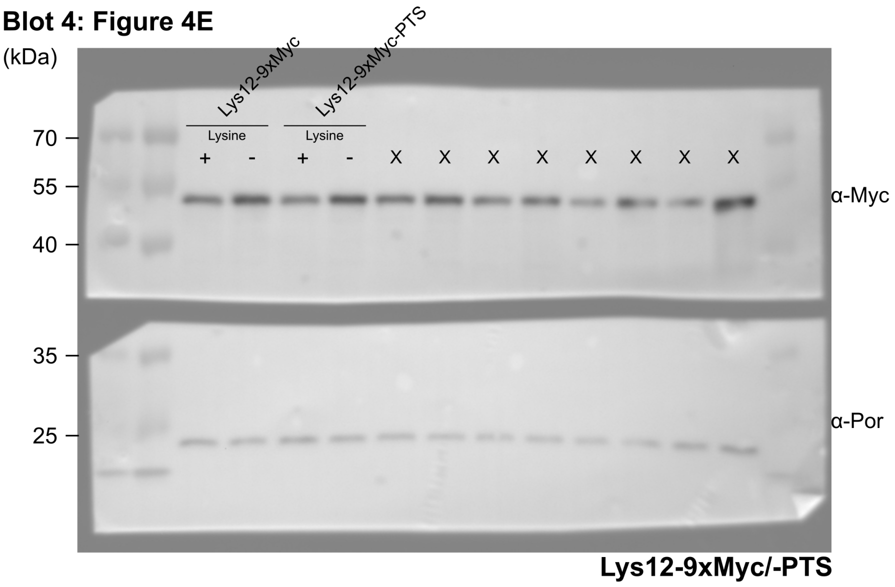

Blot 5: Figure 6C

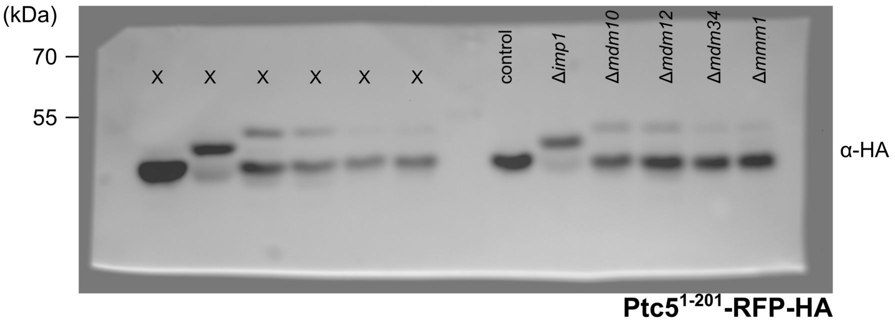

Blot 6: Figure 6F

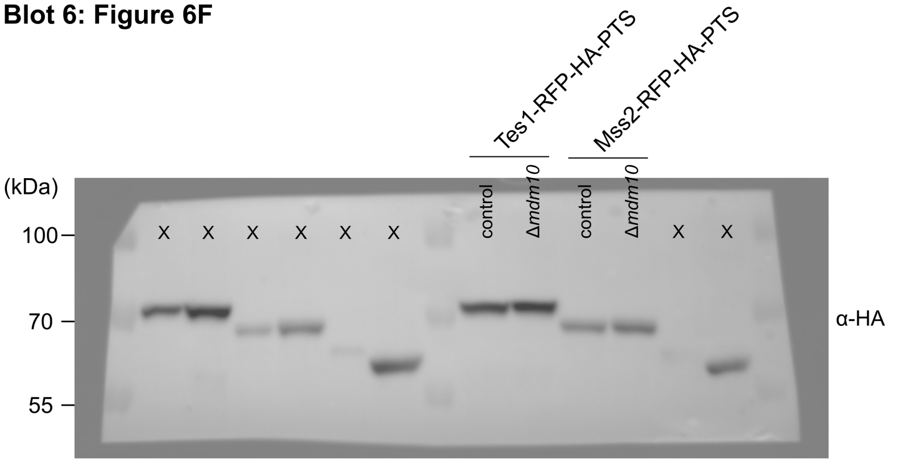

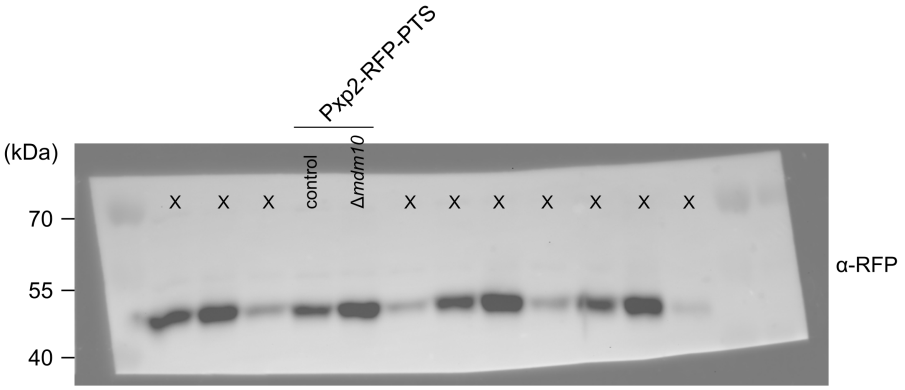

Blot 7: Figure 8D

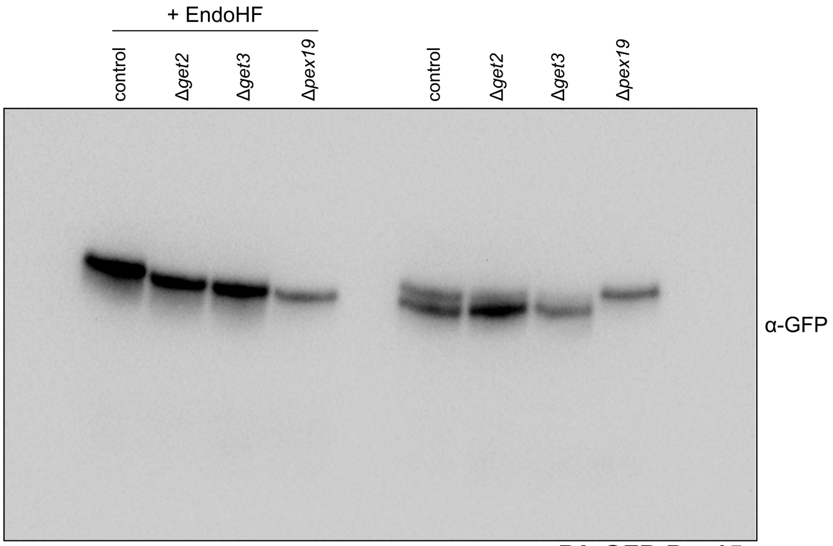

PA-GFP-Pex15g

Blot 8: Figure S2C (upper left)

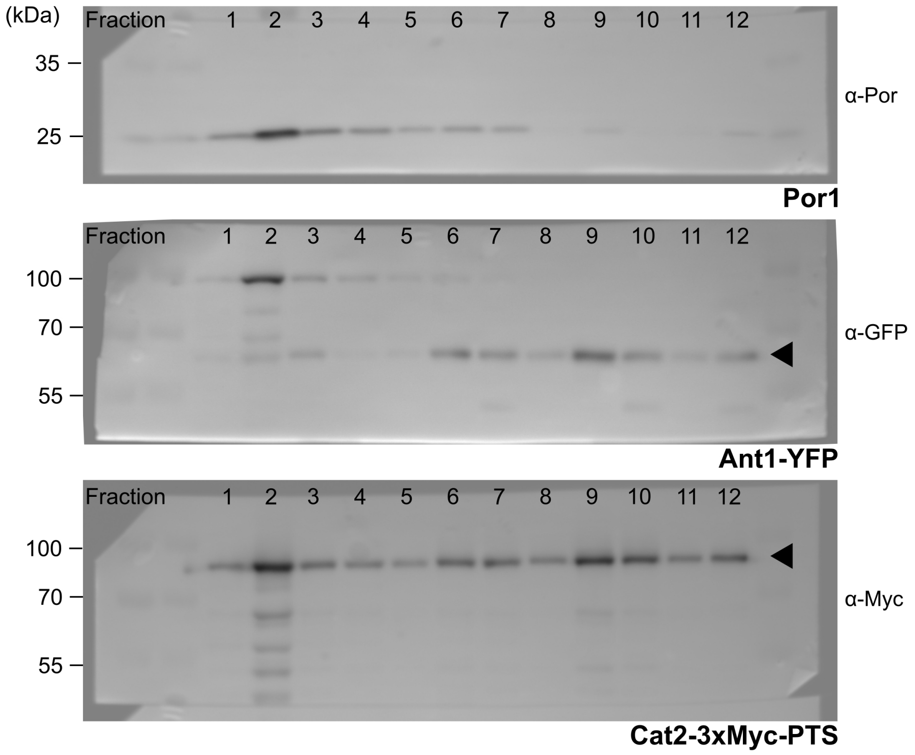

Blot 9: Figure S2C (downer left)

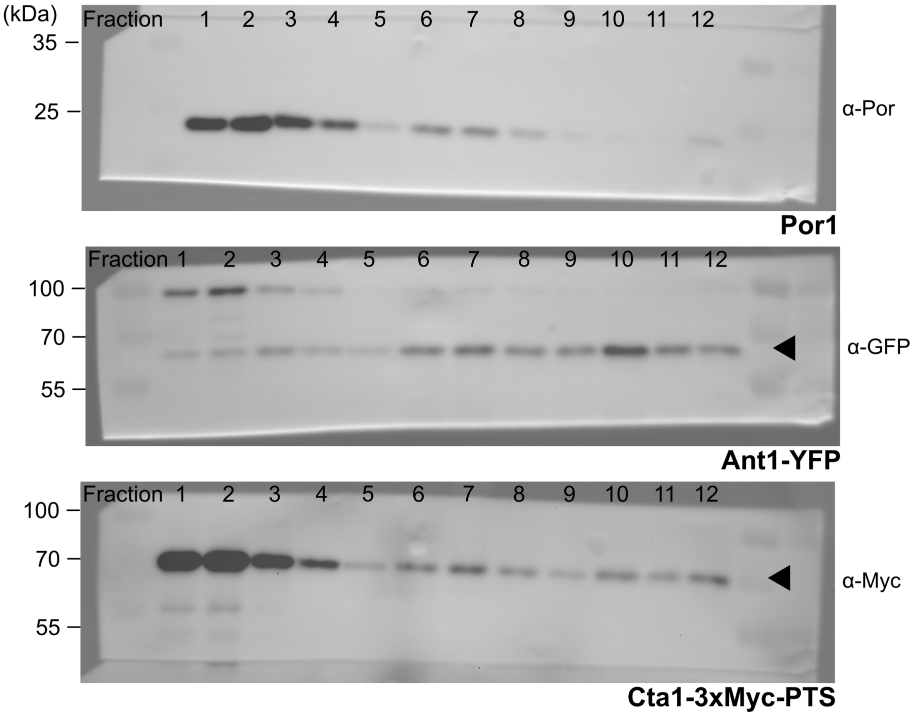

Blot 10: Figure S2C (right)

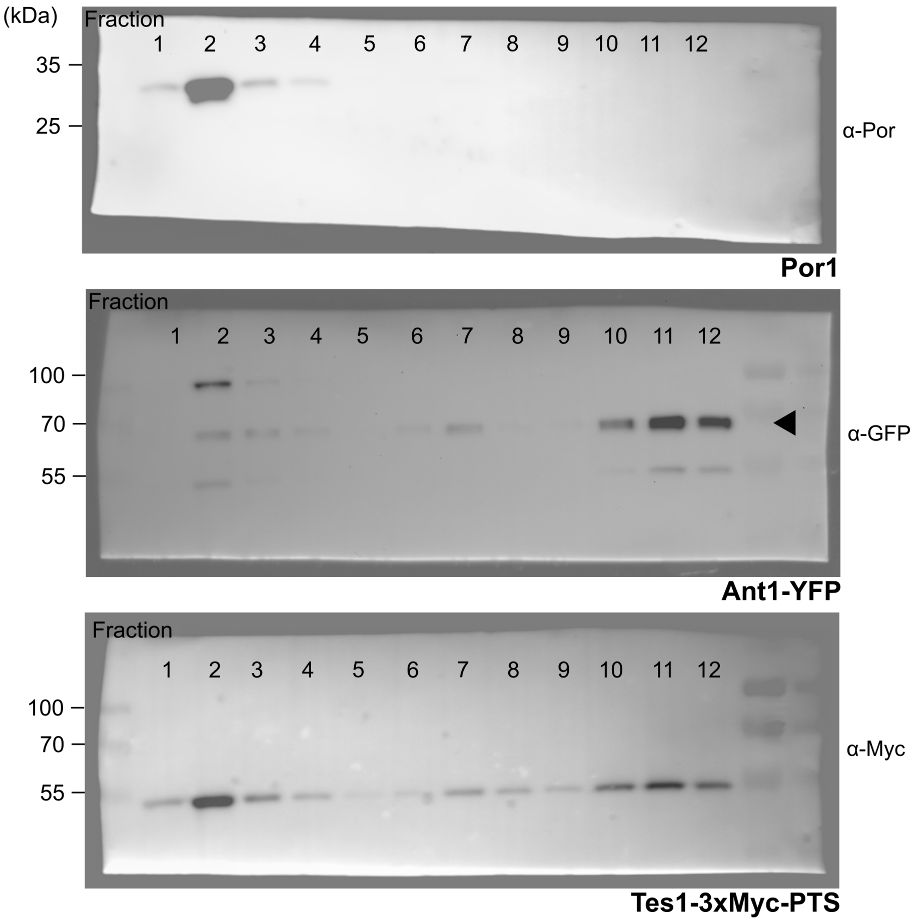

Blot 11: Figure S7B

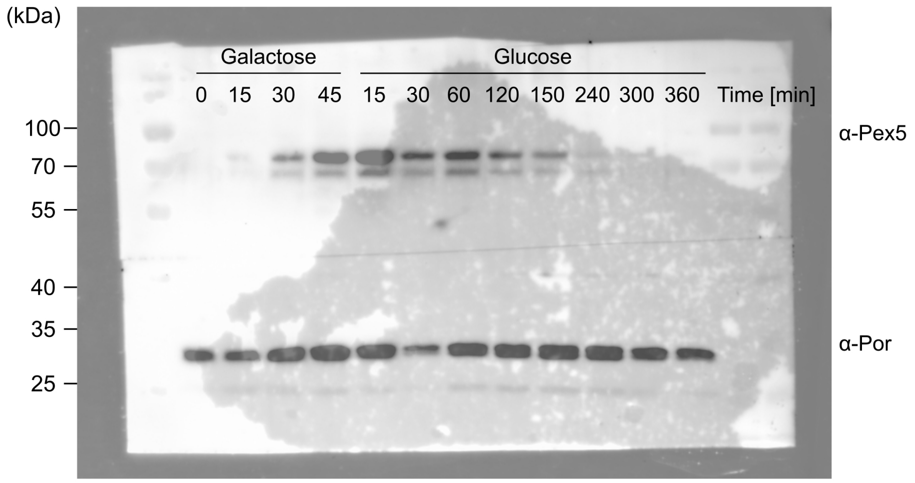

Blot 12: Figure S10A

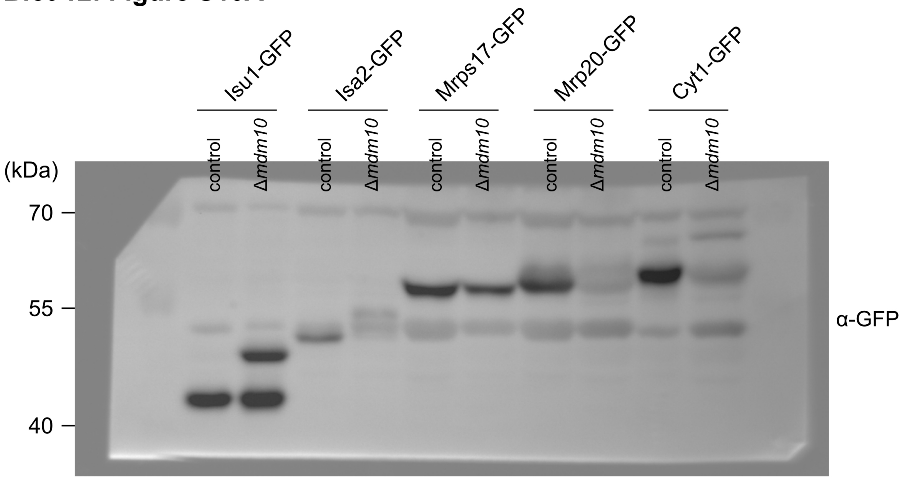

Blot 13: Figure S10C

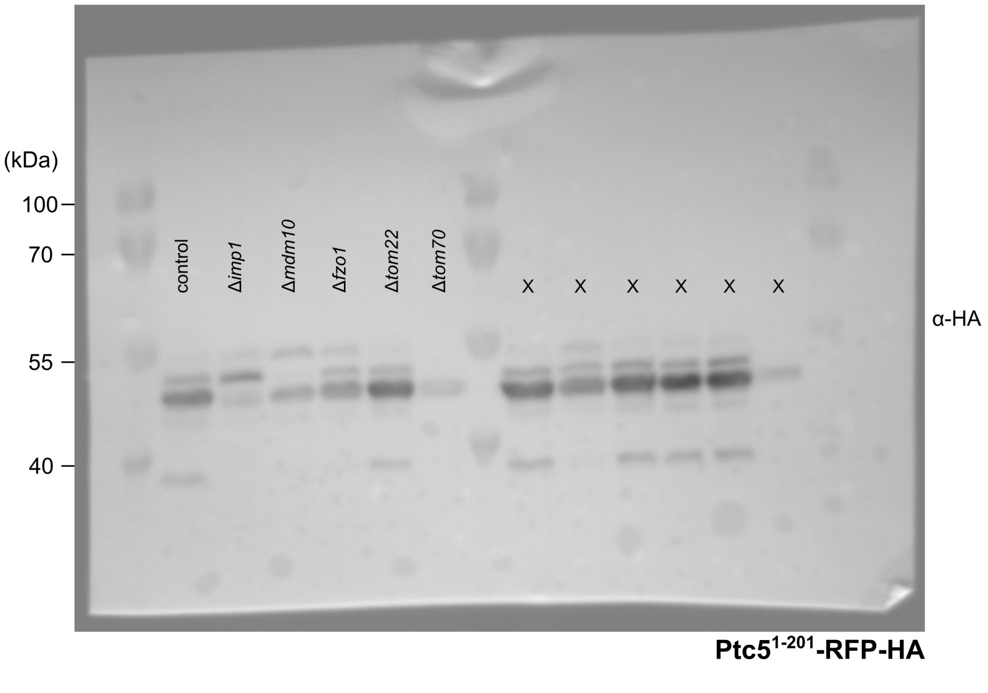

Blot 14: Figure S20B

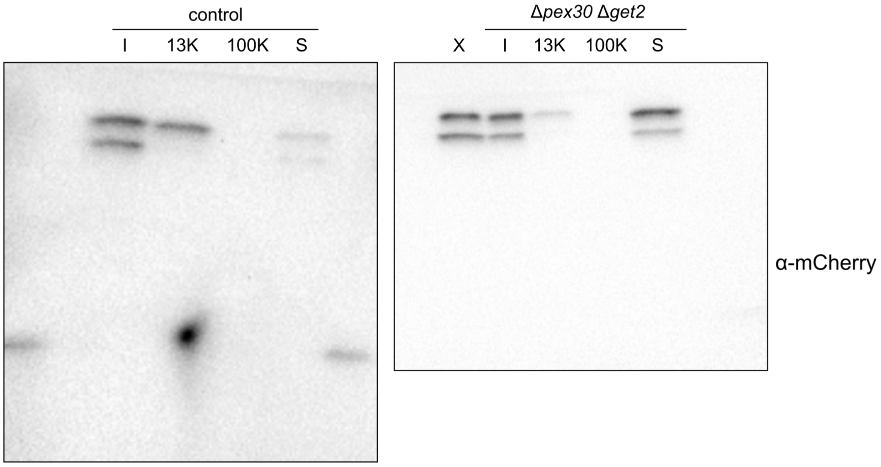

mCherry-PTS

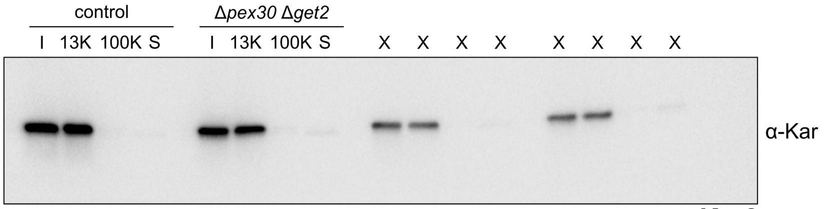

Kar2

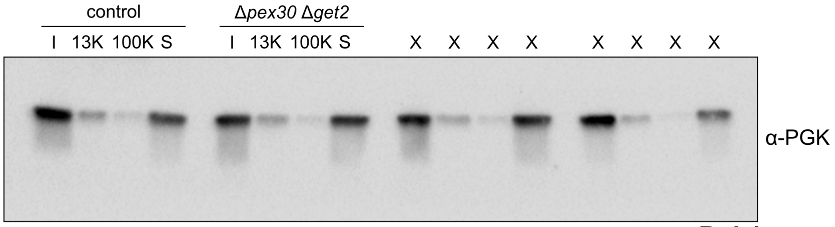

Pgk1
