# Supplementary material for: Proteins that carry dual targeting signals can act as tethers between peroxisomes and partner organelles
Source: PLoS Biol. 2024 Feb 20;22(2):e3002508. doi: 10.1371/journal.pbio.3002508 (PMC10906886; doi:10.1371/journal.pbio.3002508)
Supplement: Attachment — Submitted filename: RESPONSE_LETTER.pdf [file pbio.3002508.s029.pdf]

First, we would like to thank the editor and the reviewers for their constructive comments and their interest in our findings. We have managed to address most of the points raised by the reviewers and are confident that our paper is more convincing after revision. Please find our "point by point" responses below. We hope that we can work together towards its publication.

Response to comments from reviewer 1:

1. Overexpressing proteins with MTS and PTS1 signals only modestly increases mitochondria-peroxisome association. For example, Fig 1G shows overexpression of either of two of these proteins increases the percent of peroxisomes associated with mitochondria from about 55% to 60 or 65%. What remains unclear, however, is whether endogenous proteins play a significant role in mitochondria-peroxisome tethering and, more importantly, whether the modest changes in tethering are physiologically relevant. To address the first issue, genes encoding several the most abundant proteins containing both an MTS and PST1 should be deleted to see whether this significantly reduces mitochondria-peroxisome association. To address the second issue, it is necessary to show that tethering mediated by proteins with MTS and PTS1 signals affects mitochondrial or peroxisome function. Other evidence that the new tethering mechanism is physiologically relevant could include a demonstration that changes in the abundance of one of the tethering proteins or in the efficiency of the mitochondrial or peroxisome import machineries occurs in response to stress or a change in metabolic state.

These are excellent points. To address the relevance of tethering via dual affinity MTS-PTS proteins in a more physiological context we analyzed two enzymes of the lysine biosynthesis pathway that contain dual targeting signals. We found enhanced formation of PerMit contacts upon lysine deprivation (Fig.4F). This phenotype coincided with an induction of Lys12 expression (Fig. 4E) and was dependent on the PTS1 in Lys12 (Fig. 4G). In addition, we show in another fungus (*Ustilago maydis*, which we also used for EM) that endogenously expressed and internally tagged Um_Ptc5 accumulated at PerMit junctions upon oleic acid addition (Fig. 1F). We believe that both observations highlight the relevance of the identified mode of sorting and tethering.

2. The screen for factors involved in targeting of Ptc5 to peroxisomes is well done but it is not clear how the results "strengthen our hypothesis that the proximity of mitochondria and peroxisomes is crucial for peroxisomal targeting of Ptc5-RFP-PTS via mitochondrial transit." Mutations that affect the targeting of MTS-containing proteins to mitochondria were not found (except Som1) but should have been if the hypothesis is correct. The effects of defects in mitochondrial import should be directly tested with mutants.

Thanks for this notion. Indeed, we do not detect many mitochondrial import mutants in our screen. We now tested Δ*tom22*, Δ*tom70* and Δ*fzo1* since Fzo1 was previously shown to tether upon overexpression (PMID: 29720625). Although we observed defects in preprotein processing and protein stability (Fig. S10C), no mitochondrial retention of Ptc5 occurred in any of these mutants (Fig. S10B). Thus, it is more likely the tethering function of ERMES (direct or indirect e.g. via dual affinity proteins losing their affinity to mitochondria in Δ*mdm10*), which is responsible for the Ptc5 sorting phenotype.

In addition, if the proximity of mitochondria and peroxisomes is actually crucial for tethering, the Ptc5 targeting defects in the mutants should be suppressed by the artificial mitochondria-peroxisome tether (at least in mutants that have an intact PTS1 import pathway). This should be determined.

We agree that this is a crucial experiment. This experiment was part of the initial submission, but probably not highlighted or explained enough. To clarify - the synthetic tether can partially restore peroxisomal targeting of Ptc5 (Fig. 7F) and we have now better explained this in the text and quantified the data.

3. The various experiments with the ERMES complex are interesting, but there is no mechanistic insight into how ERMES could affect protein targeting to peroxisomes, peroxisome biogenesis, or peroxisome tethering. As the study stands now, it remains possible defects in these processes in cell lacking ERMES are indirect.

The referee is correct. Depletion of ERMES has manifold consequences and this is stated in the manuscript. However, we think that we provide some insight. ERMES affects correct targeting of dual affinity proteins. We think of ERMES as a bridge, where sorting of dual affinity proteins takes place, but to finally conclude this further research is needed. Our genetic data show that

Δ*mdm10*Δ*pex5* mutants have no additive effects on peroxisome number (Fig. S16A). The previously identified interaction of Pex11 and Mdm34 may contribute to PerMit, but Pex11 is not necessary for Ptc5 transit (Fig. S10D).

4. The experiments investigating the possible role of Pex15 in ER-peroxisome tethering are perplexing. While it is true that Pex15 can be inserted into the ER or peroxisomes, it is not clear how this could lead to tethering of the organelles. The study suggests some Pex15 transits through the ER on the way to peroxisomes. Even if this were correct (and the evidence is indirect and not convincing) it is not clear how this would increase the association of the ER and peroxisomes. A mechanism should be proposed and tested.

We addressed this type of dual targeting as previous data revealed that many of the proteins acting as tethers are dually localized (PMID: 29720625). This, together with our novel findings suggested that it might be worth considering dual targeting and tethering as a unit. Pex15 is an interesting model protein to probe these ideas as it shows up in mitochondria, in the ER and in peroxisomes depending on the genetic background and reporter construct used. We now provide additional evidence that Pex15 can transit from the ER *en route* to peroxisomes by showing that it accumulates in the ER in the absence of the ER resident ATPase Spf1 (Fig. 8E - 8F). Moreover, we show that a reporter protein consisting of the N-terminus of Pex15 attached to a mitochondrial tail anchor creates artificial PerMit contacts (Fig. 8I). Thus, there is information for tethering in the N-terminus.

5. There are similar concerns about the proposed roles of the ERMES and GET complexes in peroxisome biogenesis. The study suggests "both [complexes] are involved in regulating peroxisome growth and division by sorting of dual affinity cargo." Even if it were correct that complexes play a direct role in sorting, what is the mechanism of tethering and how does this affect peroxisome biogenesis? Some insight is necessary.

While our data for ERMES and the GET proteins clearly show that they are involved in sorting and in tethering – we agree with the reviewer that this could be either direct or indirect. All of the investigated MTS-PTS tethers change the localization pattern upon depletion of ERMES proteins (Fig. 6A, D and E). Combined depletion of Get2 and Pex30 causes severe defects in peroxisome formation (Fig. 9A). Deletion of *GET2* together with *MDM10* shows additive effects on peroxisome abundance suggesting that they act independently (Fig. 9C-D). We tried to better describe our data to make our reasoning clearer. We are confident that these data nicely showcase that we need to further address the redundancy of factors involved in peroxisome biogenesis and contact site formation.

<u>Response to comments from reviewer 2:</u>

Major points
1. This paper relies solely on fluorescence microscopy. Hence, the bar here is very high; however, most of the microscopy images are too small and low resolution, even when the "higher resolution" images are downloaded. Please correct. Arrows in the images (Fig 1B, 1D, S1 etc) are too small. In some of the figures, like Fig 2G&H or 3C, only the merged images are shown. These issues and the lack of any quantification make it difficult to judge whether the signals are colocalized/overlapped or not. Authors should increase size and resolution of the images, add single channel images, and quantify all of them.

We agree that the pictures and quantifications needed improvement and adapted huge parts of the manuscript according to your suggestions. For several figures we put the single channel images into the supplemental figures. We are grateful for your suggestions that now makes the manuscript stronger.

2. Electron microscopy and further functional assays should be performed to define the dual-targeted proteins as tethers (Eisenberg-Bord et al., Dev. Cell, 2016). Since peroxisomes and mitochondria exchange lipids and metabolites at their contact sites to facilitate metabolism, the authors should analyze whether the dual-targeted proteins affect e.g., β-oxidation, to understand their functional roles at the contact sites. Furthermore, the partitioning of peroxisomes upon cell division should be analyzed, because the reported PerMit tether Inp1 affects it.

Thank you for this helpful suggestion. We followed the advice and performed a transmission electron microscopy (TEM) study. Since working on *S. cerevisiae* peroxisomes is extremely challenging due to their low frequency, we chose to do this in another fungus (*Ustilago maydis*). *U. maydis* Ptc5 show similarity to the *S. cerevisiae* protein and we confirmed its ability to tether upon

overexpression (Fig. 1B). We also obtained TEM data an found that close connections between mitochondria and peroxisomes occur more frequently and the mean distance of the organelles decreases in response to elevated levels of Ptc5 (Fig. 1C-E). Thus, quantification of EM pictures reflected our fluorescence microscopy results. In addition, we show that internally tagged Ptc5 expressed under its endogenous promoter accumulated in foci at the interface of mitochondria and peroxisomes upon oleic acid treatment (Fig. 1F, Fig. S1D). Ptc5, hence, meets the three criteria for a tether proposed by Eisenberg-Bord et al., Dev. Cell, 2016. It occurs in foci at contact sites, elevates the number of contacts upon overexpression and there is a mechanistic rationale behind this type of tethering. We did not focus on beta-oxidation and inheritance so far as we feel that this is outside the scope of the current manuscript. However, addressing this is certainly interesting in future work. Interestingly, two of the proteins we identified in our initial screen for dual localization are involved in lysine biosynthesis. We show that lysine deprivation enhances PerMit contacts in a manner depending on the PTS1 of the MTS-PTS protein Lys12 (Fig. 4F-G).

3. As shown in Figure 3, authors screened for genes regulating peroxisomal targeting of Ptc5, which possesses a dual targeting sequence. Although the authors focused on Ptc5 because of its dual targeting, this screening system would extract genes that regulate PTS1-dependent peroxisomal localization. The authors need to investigate whether these genes regulate the localization specifically of the dual targeted proteins, or also other proteins that use PTS1.

We should have more clearly explained what we see. PTS1 import is only slightly affected by ERMES depletion (Fig. 7A). Several dual affinity proteins with unusual MTS even enrich in peroxisomes in Δ*mdm10* cells (Fig. 6E) and a variant of Ptc5 lacking the MTS is normally localized in peroxisomes under these conditions (Fig. S12C). Thus, it is not only the reduced ability to import PTS1 cargo that causes the Ptc5 retention phenotype of ERMES mutants. In addition, peroxisomal targeting of Ptc5 is restored upon introduction of the synthetic tether into Δ*mdm10* cells (Fig. 7F).

4. Related to above, authors should investigate whether the localization of proteins with PTS1 but without MTS is affected by KO of ERMES components.

Please see above.

5. The authors forced PerMit with an artificial tether, and investigate the effect on peroxisome morphology in ERMES mutants (Fig 5D-G), but not in WT cells. To determine whether PerMit contributes to define peroxisome number and size, and localization of dual-targeted protein, the analysis with the artificial tether should be performed also in WT cells.

In WT cells the artificial tether provokes enlargement of peroxisomes as well (Fig. S14).

6. Authors focus on peroxisome number and size because several mutants affects them. However, peroxisome dynamics is regulated by various processes, including biogenesis, fusion, fission, and maturation. Therefore, if authors observe one phenotype it does not mean that it results from the same process being affected. In other words, authors must assess all the processes of peroxisomal dynamics before concluding anything mechanistic.

The reviewer is correct. Nevertheless, it is interesting that every mutant lacking a targeting factor for any of the organelles we investigated showed accumulation of small and highly mobile peroxisomes, while all investigated tethers (dually localized MTS-PTS proteins, Pex15, synthetic tether) reduced peroxisome number and mobility. We are confident that this data is already a good hint for dual targeting induced contacts. While ERMES and Pex5 could act in the mitochondrial tethering module or at mitochondria-ER junctions, the GET complex and Pex30 are involved in tethering at the site of the ER and – given our genetic analysis – act independently of ERMES.

7. Since ERMES regulates mitochondria-ER contact sites, authors should examine how a mito-ER artificial tether affects PerMit, the localization of dual-targeted protein, and peroxisome size and number.

A synthetic mito-ER tether does not rescue the peroxisome specific phenotypes of *mdm10* cells (Fig. S12A).

Minor points
1*. Authors developed the auxin-dependent Pex13 depletion system, as shown in Fig 2E&F, to analyze the effect of Pex5/13-dependent peroxisomal protein import. However, with this system, it is not clear whether peroxisomal import is functional without auxin and it is inhibited by auxin*

treatment, because of the low resolution of the images. The authors should quantify the colocalization to confirm it.

We added the data as requested and it can now be found in Fig. 3G. Pex13-AID does affect import. This effect is significantly enhanced upon addition of auxin.

2. To test for statistical significance among more than 2 conditions, authors should not use Student's t-test, to avoid type I error.

Thank you for this notion. We corrected the tests where appropriate.

3. Images shown in Fig 2G&H are exactly same as Fig S3A&B. And the graphs in Fig 2G&H are same as left half of the graph in Fig S3A&B. Authors should eliminate duplicated data.

We apologize for this oversight and have now adapted the figures.

4. In this manuscript, the colocalization of the organelles is evaluated based on Pearson's correlation coefficient (PCC). Since PCC is largely affected by outliers, Manders' coefficient should be used.

In the colocalization data analyzed via PCC we don't have many outliers. Thus, we kept the quantifications.

Response to comments from reviewer 3:
Stehlik et. al., report that proteins that are dually targeted to peroxisomes and mitochondria, due to presence of intrinsic signal sequences, regulate membrane contact between peroxisomes and partner organelle. The proposed model is interesting however, it lacks support from the data the authors provide. While they provide some evidence to support that dual targeting occurs at contact sites their findings to claim that protein import contributes to the maintenance of contact sites are weak. The manuscript in the current form is poorly written and not organized, very difficult to understand as they do not explain the experimental setup as well as their observations before concluding their findings.

We apologize that our paper was difficult to understand. We added clearer explanations to several chapters and hope that the paper is easier to read in its current form.

Authors use peroxisome number and appearance as the only read out for protein sorting and inter-organelle contact.

We were surprised about this comment as we use different read-outs for contact. Besides reduction of peroxisome number we quantify peroxisomes adjacent to mitochondria for many examples and have expanded this analysis in the updated paper (Fig. 1, Fig. 2, Fig. 3, Fig. 4 and Fig. 7). In addition, we provide movies showing enhanced peroxisome motility upon depletion of sorting machinery. Moreover, we have now added "direct" evidence for contacts using electron microscopy (Fig. 1C-E). We believe that these four methods together are the gold standard for proving contact formation.

To support these claims and overall data especially in Figure 1, 2A and 5 (considering the limit of resolution of fluorescence microscope), the authors must provide high resolution electron microscopy images.

EM of PerMit contacts is difficult to obtain in yeast (e.g. PMID: 29720625). Thus, we needed a workaround to address this very important point. We now provide an EM study of *Ustilago maydis* cells, in which Ptc5 sorting seems to be conserved (PMID: 32398688). We verified that our quantifications of fluorescence microscopic images are likely to report on tethering (Fig. 1D-E). Moreover, we describe regulated tethering in the updated version of the manuscript (Fig. 4E-G). We believe that with this data our paper is more convincing.

Introduction:
"In mammalian cells, proximity of peroxisomes and the ER is achieved through a pair of membrane-associated proteins assisting lipid transfer [24,25]" -
Mention which proteins.

This was changed accordingly.

Figure 1:
"Overexpression of Pxp2-RFP-PTS and Cat2-RFP-PTS reduced the total peroxisome number (Fig. 1D and 1E). Both results may be explained by a potential tethering function. "
Figure reference incorrect. How does this result explain potential tethering function?

We thank the reviewer for spotting this mistake. It is fixed now.

"Overexpression of Pxp2-RFP-PTS and Cat2-RFP-PTS reduced the total peroxisome number. "
How does this support your model? Shouldn't the peroxisome number be more with increased pex-mito tethering.

One consequence of the dual affinity protein sorting can be longer retention at the contact site, which may explain the decrease in peroxisome number. This effect was reported for a PerMit reporter (PMID: 29720625), which can act as artificial dual affinity protein upon assembly or observed for the artificial tether created in the course of our present study (Fig. 7D-F). Therefore, it is one, albeit certainly indirect read out to confirm organelle tethering via dual affinity proteins.

Controls such as overexpression of proteins targeted to either only peroxisome or only mitochondria are missing.

This is an excellent suggestion. We expressed Pxp2 with an N-terminal RFP tag to mask the MTS. We did not detect increased tethering (Fig. 2H). A control experiment without PTS1 is depicted in Fig. 2C for Cat2. For Pxp2 we have published this previously (PMID: 32398688).

Better separation of organelles in density gradient is required to confirm subcellular localization.

We agree with the reviewer that for complete proof of localization a better separation may be needed. However, our purpose was to show accumulations in “non-mitochondrial” fractions and for this the gradients are informative. Especially fractions 10 and 11 are enriched in peroxisomal proteins and almost devoid of Por1 (Fig. 2F and S2C).

"We then utilized overexpressed internally Myc-tagged versions of two candidate proteins integrated into the LEU2 locus: Cat2 and Pxp2. "
It's unclear how these strains were generated.

Plasmids were integrated into the LEU2 locus via homologous recombination. The Myc-tag resides upstream of the C-terminal dodecamer containing the PTS1. We have now added this information to the materials and methods section.

Is it Cat2-RFP-PTS or Cat2-RFP-HA-PTS?

We apologize for this inaccuracy. It is a Cat2-RFP-HA-PTS. We have now fixed this also in the manuscript body.

"Thus, overexpression of proteins that have a mitochondrial and a peroxisomal targeting signal can enhance the formation of contacts between both organelles."
Authors must provide high-resolution electron microscopy images.

Please see above.

Figure 2:
Why Pex3-GFP is used as a peroxisome marker? Is this a good marker to count peroxisome number especially because Pex3 is also localized in the ER membrane?

Pex3-GFP is an established marker and we, as well as many others in the field, regularly use it. Importantly, tagging of Pex3 does not interfere with peroxisome biogenesis. In addition, we have obtained similar data for Pex14 fused to mNeonGreen (Fig. S3B and C)

Figure 2A- More Per-Mit contact are seen in pex5 mutant as compared to WT in the representative image provided. Single channel images should be provided. EM images might help. Including ER marker might help.

We have changed the picture. We were not able to obtain EM data for *Saccharomyces cerevisiae* (please see above). However, in aggregate with all the new data (Fig. 1; Fig. 2 and Fig. 4) as well as EM in *Ustilago maydis* (Fig. 1) – we have strong support to our hypothesis.

Figure 2J: In the legend it is mentioned that the quantification in J is of the strain described in H. Is that correct?

Many thanks for spotting this error. It is corrected.

Figure 2H: Why is Cat2-RFP-PTS still targeted to peroxisomes after depletion of Pex13-AID-HA. Please provide single channel images.

Since the depletion only occurs from addition of auxin and since Pex13 is functional prior to its elimination, this is likely protein produced prior to Pex13 depletion. Please note that mitochondrial targeting of Cat2 is not affected in this mutant, thus cytosolic background is not expected.

Figure 4: Include non ERMES mutant such as mitochondrial fusion mutant as a control.

We added data for Δ*fzo1* (lacking mitofusin1) and for import mutants (Δ*tom22* and Δ*tom70*). None of these mutants affected translocation of Ptc5 (Fig. S10B). Thus, ERMES appears to have a specific function for transit of Ptc5.

Figure 5: What is PTC-RFP punctae that do not colocalize with Pex3-GFP? Include Pex11 mutant as a control.

These are mitochondria of ERMES mutants (please see other panels showing cells with Mito-Marker). Depletion of Pex11 does not interfere with Ptc5 sorting (Fig. S10D).

Figure 6:
"Prolonged overexpression of PA-GFP-Pex15g resulted in peroxisomes attached to the ER (Fig. 6F) resembling peroxisomes attached to mitochondria by overexpression of dual affinity PTS1 proteins (Fig. 1)." Insufficient data to conclude that. Include get mutants in 6F.

To strengthen our point that Pex15 acts as a tether protein, we forced it into mitochondria. This increased tethering to this organelle. This new data is now shown in Figure 8I.

Figure 6A: Including an ER marker might help.

We have added an ER marker to several panels.

Figure 7: "Cells with intact mitochondria showed the lowest number of peroxisomes, however peroxisomes that remained frequently in proximity to mitochondria (Figs 7A and S8). "
If that's the case, then why Pex30 mutant exhibits more peroxisomes with intact mitochondria.

According to our model this behavior is expected. In Δ*pex30*Δ*get2* mutants, peroxisomes and (maybe other organelles) will tend to cluster at mitochondria as proteins with multiple targeting signals are likely to enrich (mis-localize) at this organelle (e.g. Schuldiner et al. 2008, Cell; our data on Pex15), which in turn will recruit the organelle of final destination. Peroxisomes depend on factors shared with both organelles given the strong phenotype of a Δ*get2*Δ*mdm10* strain (Fig. 9C-D). We think that the phenotypes of double mutants are interesting for readers as they show how depletion of multiple contact site proteins or targeting factors affect organelle homeostasis. Therefore, we like to keep this part of the paper.

"These data are consistent with the hypothesis that ERMES and GET complex exhibit a similar role - both are involved in regulating peroxisome growth and division by sorting of dual affinity cargo." Insufficient data to conclude this. Suggest authors to remove this section on synthetic genetic interactions from the manuscript.

We softened this statement and hope that this now more appropriately represents our data. However, since our data suggest that protein transit, contact site formation, peroxisome growth and proliferation are all connected – we think that these issues should be addressed together. Hence, we now state: "Both regulate contacts and distribution of dual affinity cargo". Dual-affinity proteins tend to cluster at contact sites (e.g. Fig. 1F and Fig. 7). If they are there they can tether.
