# Supplementary material for: Proteins that carry dual targeting signals can act as tethers between peroxisomes and partner organelles
Source: PLoS Biol. 2024 Feb 20;22(2):e3002508. doi: 10.1371/journal.pbio.3002508 (PMC10906886; doi:10.1371/journal.pbio.3002508)
Supplement: Attachment — Submitted filename: RESPONSE.docx [file pbio.3002508.s031.docx]

Dear editors, dear reviewer, many thanks for evaluating our paper und your many helpful suggestions.

Academic Editor's comments

Lines 340-342:
"Upon depletion of the previously identified PerMit tether Pex34, we visually observed more mitochondrial localization (Fig 5C and S8 Fig), however, we did not observe a difference in quantifications (Fig 5D), speaking for redundant modes of tethering."

This comment is very unclear: either there is a phenotype and it is quantifiable, or there isn't. It seems you are trying to say that you saw a phenotype, but the quantification says the opposite. Perhaps it would be better to just remove the comment.

We appreciate this point. We see more mitochondrial staining upon visual inspection. Nevertheless, the PCC is identical. This may result from the different peroxisome morphology in ∆*pex34* mutants, but we left out the comment to avoid any confusion.

Reviewers' comments

Rev. 2:
The authors have addressed some of my concerns, but did not analyze the functional role of the contacts and answered textually to some questions that I believed were to be addressed experimentally. I therefore suggest that the paper is further processed for publication, provided that the authors tone down some of the conclusions. For example, in the discussion I would avoid using the term tethers (because they did not demonstrate that these proteins act as tethers) and resort to the same wording used in the abstract.

We toned done the first statement in the discussion section and left the abstract as it is. We switched to the title suggested by the PLoS biology team, which includes the term tethers. We hope that this is justified given our EM data and further microscopic data

Moreover, the term "regularly" in the first paragraph of the discussion implies that this is the case in most instances, but this is far from being demonstrated. I would consider using the word "often" that is perhaps more appropriate.

The reviewer is correct. We have changed the term.

Please expand the discussion of whether ERMES might affect proximity also between ER and mitochondria not as a physical tether, but because of its role in protein sorting and import. Refer also to the recent cryoEM structure by Wozny et al of ERMES PMID: 37165187.

We added this interesting reference (lines 620-24).

Other comments
1. Please correct the term "mitofusion 2" into Mitofusin 2

2. when discussing the dual targeting of Mitofusin 2, please refer to the recent (2023) publication by Naon et al describing the mitofusin 2 splice variants that are targeted to the ER to tether it to mitochondria PMID: 37347868.

3. introduction, line 58: "transfer of molecules", please add "and ions" as contact sites between ER-PM and ER-mitochondria are also crucially involved in the transfer of Ca2+

All these points have been addressed.
